# Ubiquitin E3 Ligases and p53 in Doxorubicin-Induced Cardiotoxicity

**DOI:** 10.3390/ijms262110252

**Published:** 2025-10-22

**Authors:** Shingo Tachibana, Yoichiro Otaki, Jun Goto, Tetsu Watanabe, Masafumi Watanabe

**Affiliations:** Department of Cardiology, Pulmonology, and Nephrology, Yamagata University School of Medicine, Yamagata 990-9585, Japan; stachibana@med.id.yamagata-u.ac.jp (S.T.); j-goto007008@med.id.yamagata-u.ac.jp (J.G.); tewatana@med.id.yamagata-u.ac.jp (T.W.); m-watanabe@med.id.yamagata-u.ac.jp (M.W.)

**Keywords:** ubiquitylation, E3 ligase, p53, Doxorubicin-induced cardiotoxicity

## Abstract

Doxorubicin (Dox) is a widely used anti-cancer drug. It has proven efficacy against various cancers, although the clinical application of Dox has been limited due to dose-dependent, irreversible, and fatal Dox-induced cardiotoxicity (DIC). The mechanism of DIC remains unclear. p53 plays a key role in DIC via cardiomyocyte loss due to cell death and oxidative stress. Its expression is strictly controlled by post-translational modifications, and its suppression in cardiomyocytes reportedly ameliorates DIC. The ubiquitin system regulates biological processes that are fundamental to the development of cardiovascular diseases. The dysregulation of several ubiquitin E3 ligases is reportedly associated with DIC development through the upregulation of p53. Ubiquitin E3 ligases are classified into four groups; all classes of E3 ligases are involved in p53 degradation. In this review, we focus on recently emerging topics regarding the role of E3 ligases in the regulation of p53 degradation. We also provide an overview of the functional roles of E3 ligases in DIC. Recent reports have identified cardioprotective agents for DIC through ubiquitin E3 ligase-mediated p53 suppression. Here, we present some findings regarding the current development of cardioprotective agents for DIC. These agents may serve as a novel therapeutic target for the treatment of DIC.

## 1. Doxorubicin Cardiotoxicity and p53

Doxorubicin (Dox) is a secondary metabolite of a mutant strain of *Streptomyces peucetius* var. *caesius*. Dox is an inhibitor of DNA topoisomerase II, which induces DNA damage in cancer cells [1,2]. Dox is an anthracycline anti-cancer drug applied in treating various cancers [3,4]. However, its clinical utility is limited due to its well-recognized dose-dependent cardiotoxicity [5]. In adults, the risk of Dox-induced cardiotoxicity (DIC) increases markedly when the cumulative dose exceeds 400–700 mg/m^2^, although cardiotoxicity can occur at lower doses [6]. DIC is characterized by a decrease in left ventricular contractility and global longitudinal strain, a potential early diagnostic parameter [7,8].

Cancer survivors remain at an increased risk of cancer therapy-related cardiac dysfunction for years or even decades after treatment completion. This highlights the importance of long-term cardiovascular follow-up [7]. Therefore, Dox treatment poses a lifelong health risk, even in patients without obvious DIC, underscoring the importance of mitigating the cardiotoxic effects of Dox. The pathology of DIC is complex and is reportedly associated with oxidative stress, inflammation promotion, apoptosis induction, ferroptosis, mitochondrial impairment, endoplasmic reticulum stress, autophagy pathway dysregulation, epigenetics, and fibrosis. However, cardiomyocyte loss due to cell death and oxidative stress is considered the main cause [1,8]. Cardiomyocyte death reportedly results from apoptosis and ferroptosis in DIC [9].

Apoptosis refers to programmed cell death. Dox triggers cardiomyocyte apoptosis via excessive oxidative stress and mitochondrial damage. In addition, by inhibiting topoisomerase II, Dox stabilizes the covalent complex between cleaved DNA and topoisomerase II, inhibiting recombination after DNA breaks. This eventually induces apoptosis caused by DNA double-strand breaks [2]. Ferroptosis is a newly recognized regulatory cell death process. It is characterized by the accumulation of iron and lipid reactive oxygen species (ROS). Under steady conditions, solute carrier family 7 member 11 (SLC7A11) promotes cysteine uptake and intracellular glutathione synthesis to guarantee the anti-lipid peroxidation function of glutathione peroxidase 4 (GPX4). Doxorubicin downregulates these key proteins and promotes ferroptosis by increasing intracellular iron accumulation and driving lipid peroxidation in cardiomyocytes [10,11]. Mitochondrial iron and ROS accumulate, further exacerbating cellular injury [12].

p53 is a critical protein that induces apoptosis and ferroptosis in response to DNA damage and stress, respectively. Mechanistic studies have demonstrated that p53-dependent pathways exist, including those involving metabolic alterations [13], autophagy [14], apoptosis [15], and ferroptosis [13,16,17,18]. Accumulating evidence has shown that p53 is activated in Dox-treated hearts and cardiomyocytes. Increased p53 protein levels are observed early after Dox exposure, often without the corresponding upregulation of TP53 mRNA [18,19]. p53 is modulated by several post-translational modifications, such as phosphorylation, ADP-ribosylation, acetylation, and ubiquitylation, which interact with each other in a network [20]. The phosphorylation of p53 occurs primarily at serine and threonine residues at the N- and C-termini and is triggered by the DNA damage response [21]. The phosphorylation of Ser15 inhibits binding to the mouse double mutant 2 homolog (MDM2) and promotes p53 stabilization and transcriptional activation [22]. The ADP-ribosylation of p53 is primarily catalyzed by poly (ADP-ribose) polymerase-1 (PARP1) and occurs within its DNA-binding and C-terminal domains. This modification regulates the transcriptional activity and DNA-binding capacity of p53 and functions as a critical feedback mechanism in DNA damage response and cell fate determination [23]. The acetylation of p53 enhances its transcriptional activity and is essential for DNA damage response [24]. p53 has multiple acetylation sites at its C-terminus and DNA-binding domain that are site-specifically acetylated by various enzymes [25]. Ubiquitylation is an important reaction that regulates p53 levels. MDM2 primarily regulates p53 by attaching multiple ubiquitin chains to p53 and promoting its degradation by the proteasome [26]. The mono-ubiquitylation of p53 is involved in nuclear export [27]. Dox reportedly induces p53 activation and stabilizes it in cardiomyocytes by markedly inhibiting its ubiquitin-dependent proteasomal degradation [28]. Therefore, we investigated the role of E3 ligases, which are the main components of this post-translational modification.

## 2. Ubiquitin E3 Ligase

Ubiquitylation plays a pivotal role in a wide range of cellular processes such as signal transduction, transcriptional regulation, and the maintenance of homeostasis [29]. Ubiquitin is a 76-amino-acid protein with a molecular weight of approximately 8.5 kDa. It was first identified in the bovine thymus in 1975 [30]. The ubiquitylation of substrate proteins can lead to their degradation via the proteasome or lysosome, induce changes in their subcellular localization, or modulate their functional properties [31]. The ubiquitylation process is orchestrated by a cascade of three enzymes, E1 (activating), E2 (conjugating), and E3 (ligating), along with various scaffold proteins. While the human genome encodes only a limited number of E1 enzymes and a moderate number of E2 enzymes, it gives rise to a highly diverse set of over 600 putative E3 ligases and E3 ligase complexes. This reflects their critical role in substrate specificity [32]. E3 ligases are categorized into four major classes based on their domain structure and catalytic mechanism: really interesting new genes (RINGs), homologous to the E6AP C-terminus (HECT), RING-between-RINGs (RBRs), and U-box.

### 2.1. RING-Type E3 Ligases

RING-type ligases are the most abundant class of E3 enzymes found in the human genome. They function as molecular scaffolds that simultaneously bind to both the E2 enzyme and the substrate, facilitating the direct transfer of ubiquitin from E2 to the substrate without forming a covalent intermediate. The RING domain, which is characterized by a C3HC4 zinc finger motif, is essential for E2 binding and catalytic orientation. Representative examples include MDM2, c-Cbl, and Pirh2, and many RING-type E3 ligases are involved in regulating key cellular processes such as the cell cycle, apoptosis, and DNA repair [33].

### 2.2. HECT-Type E3 Ligases

HECT E3 ligases employ a two-step mechanism. First, the ubiquitin moiety is transferred from the E2 enzyme to an active-site cysteine residue in the HECT domain of the E3 ligase, forming a transient E3–Ub thioester intermediate. Ubiquitin is then covalently attached to a lysine residue on the substrate [34]. This mechanism provides additional regulatory control over substrate modifications. Prominent members of this class include E6AP, neural precursor cell-expressed developmentally downregulated protein 4 (NEDD4), and HECT and RLD-containing E3 protein ligase (HERC). These enzymes are often implicated in oncogenesis, viral infection, neural maldevelopment, and cardiac disease [35].

### 2.3. RBR-Type E3 Ligase

RBR ligases represent a hybrid class that shares structural features with both RING and HECT ligases. These enzymes contain two RING-like domains (RING1 and RING2) separated by an intermediate RING (IBR) motif. Functionally, RING1 recruits E2, whereas RING2 (also called Rcat) forms a thioester intermediate with ubiquitin, similarly to HECT-type ligases. This sequential ubiquitin transfer ensures specificity and flexibility. A notable example is Parkin, a protein central to mitochondrial quality control that is mutated in familial Parkinson’s disease [36].

### 2.4. U-Box-Type E3 Ligase

U-box-type E3 ligases form a distinct subclass characterized by a U-box domain, a modified RING-like fold lacking canonical zinc-chelating residues. Structural stability is maintained by hydrogen bonds and salt bridges rather than zinc coordination, enabling E2 recruitment and ubiquitin transfer [37]. Like RING-type ligases, they catalyze direct ubiquitin transfer from E2 to the substrate without forming an E3–ubiquitin intermediate [38]. Representative members include the C-terminus of the Hsp70-interacting protein (CHIP), PRP19, and UBE4B, which are implicated in protein quality control, stress responses, and diverse cellular processes.

## 3. Ubiquitin E3 Ligases Associated with p53 Protein Expression

**Figure 1:**
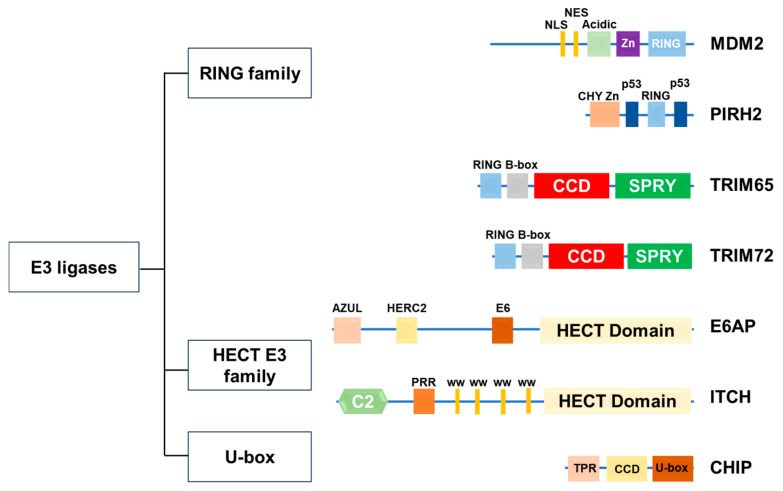
Structure of E3 ligases involved in p53 degradation in heart. NLS, nuclear localization signal; NES, nuclear export signal; Acidic, acidic domain; Zn, zinc finger domain; RING, ring finger domain; CHY Zn, CHY zinc finger domain; p53, p53-binding domain; CCD, coiled-coil domain; SPRY, SPRY domain; AZUL, AZUL-binding domain; HERC2, HERC2-binding domain; E6, E6-binding domain; C2, C2 domain; PRR, proline-rich region; WW, WW domain; TPR, tetratricopeptide repeat domain; U-box, U-box domain.

As shown in Table 1, recent experimental studies demonstrated the role of ubiquitin E3 ligases in DIC development through the regulation of p53 expression (Table 1). The structures of these E3 ligases are shown in Figure 1.

### 3.1. MDM2

MDM2 is a 56 kDa protein originally identified as a product of the mdm-2 gene, which is amplified in certain mouse tumor cell lines [39]. MDM2 consists of a p53-binding domain, acidic domain, p300-binding domain, zinc finger domain, and ring finger domain. MDM2 is primarily localized in the nucleus but also possesses nuclear localization and export signals, which are important for MDM2 to regulate p53 in the nucleus [40]. MDM2 directly binds to p53 and inhibits its transcriptional activity, whereas p53 promotes MDM2 transcription. MDM2, in turn, functions as an E3 ubiquitin ligase that ubiquitinates and targets p53 for proteasomal degradation [41]. This reciprocal regulation forms a negative feedback loop that maintains low p53 protein levels under physiological conditions [42,43]. In certain cancers, MDM2 overexpression leads to functional p53 inactivation [44,45,46,47], and MDM2 inhibitors have been investigated as potential p53-activating anti-cancer agents [48,49,50,51,52,53]. Regarding the relationship between MDM2 and heart disease, it is known that MDM2 dysfunction promotes p53 activation and impairs oxidative stress control and mitochondrial metabolism. These changes cause pathological myocardial hypertrophy, reduced left ventricular function [54], and cardiac dysfunction due to abnormal β-adrenergic receptor signaling [55]. Research indicates that the abnormal activation of MDM2 reduces HIF1α/HIF2α in hypertrophic cardiomyopathy model mice, causing microcirculatory disorders [56]. In the context of DIC, a KEGG pathway analysis of transcriptomic data from human iPSC-derived cardiomyocytes exposed to Dox revealed the upregulation of the p53 signaling pathway, including MDM2 [18]. Previous reports have shown that p300, a transcriptional coactivator required for maintaining the differentiated phenotype of cardiac myocytes, functions as an E4 ligase. It also regulates p53 expression via MDM2-dependent ubiquitin proteasomal degradation in the heart [28,57]. Transgenic mice overexpressing p300 in the heart exhibit a higher survival rate and preserved left ventricular function compared to wild-type mice in DIC. The overexpression of p300 in cardiomyocytes suppresses the Dox-mediated increase in p53 levels and subsequent apoptosis through MDM2 upregulation [28,57]. These findings support the importance of MDM2-mediated p53 degradation in DIC.

### 3.2. Pirh2

The p53-induced RING-H2 protein (Pirh2), also known as RCHY1, is a RING finger-containing E3 ubiquitin ligase that plays a crucial role in regulating the turnover of proteins involved in tumorigenesis and cellular stress responses. Pirh2 was originally identified as a p53-inducible gene that promotes the ubiquitin-mediated proteasomal degradation of p53 [58]. Pirh2 is expressed in multiple tissues, including the liver, brain, lungs, and testes. It exists as five isoforms (Pirh2A-D and Pirh2B) generated via alternative splicing [59]. Pirh2 has a molecular weight of approximately 40 kDa and features several functionally important domains, including a RING finger domain, CHY zinc finger domain, and nuclear export signal.

Pirh2 has a well-characterized role in modulating the stability of tumor suppressor proteins and cell cycle regulators including p53, p63, p73, c-Myc, and p27Kip1 [60]. It is involved in the DNA damage response, apoptosis, cell cycle progression, and epithelial–mesenchymal transition. In particular, Pirh2 ubiquitinates and degrades p53, independent of its phosphorylation status at Ser15, which distinguishes it from MDM2.

Recent studies have suggested that androgen-induced gene 1 (AIG1) is involved in ferroptosis in DIC [61]. This study demonstrated that AIG1 protects cardiomyocytes from Dox-induced ferroptosis and cardiotoxicity by directly promoting the ubiquitylation and degradation of p53 via direct binding to Pirh2.

### 3.3. TRIM65

Tripartite motif (TRIM) family proteins, most of which have E3 ubiquitin ligase activity, have various functions in cellular processes, including intracellular signaling, development, apoptosis, protein quality control, innate immunity, autophagy, and carcinogenesis [62]. Currently, more than 80 TRIM genes are known to exist in humans. Most TRIM family proteins are ubiquitin E3 ligases because they contain a RING finger domain. TRIM proteins are characterized by an N-terminal region containing one RING finger domain, one or two zinc domains named B-boxes (B1 and B2 boxes), and an associated coiled-coil region. TRIM proteins are classified into 11 subfamilies, based on their domain organization. The variability in the C-terminal domain facilitates the multifunctionality of TRIM proteins, which are pivotal for regulating intracellular signaling and transcription, innate immunity, autophagy, and tumorigenesis. Accumulating evidence indicates a crosstalk between the TRIM protein family and p53 in cancers [63,64].

Human TRIM65 is a 517-amino-acid protein originally identified as a gene with single-nucleotide polymorphisms (SNPs) associated with cerebral white matter lesions [65,66]. TRIM65 was later shown to be a cofactor in the regulation of miRNA function [67]. TRIM65 is composed of RING, B-box, coiled helix, and SPRY domains [68]. TRIM65 reportedly targets p53, annexin A2 (ANXA2), Axin1, Rho GTPase-activating protein 35 (ARHGAP35), Trinucleotide repeat-containing 6 (TNRC6), Melanoma differentiation-associated protein 5 (MDA5), and Vascular cell adhesion molecule 1 (VCAM1) for ubiquitylation [67,69,70,71,72,73,74,75]. TRIM65-mediated p53 ubiquitylation and degradation can directly inhibit apoptosis, reduce autophagy flux through the classical mTOR signaling pathway, and eventually promote carcinogenesis in cervical cancer [76]. A recent study demonstrated that cardiac-specific TRIM65 overexpression ameliorates cardiac dysfunction and remodeling in DIC, together with an increase in SLC7A11 and GPX4. TRIM65 facilitates the ubiquitylation and subsequent degradation of p53, thereby mitigating DIC by inhibiting ferroptosis in cardiomyocytes. Therefore, TRIM65 may be a promising target for DIC treatment [77].

### 3.4. TRIM72

TRIM72, also known as Mitsugumin-53, was discovered through protein library screening in 2009 [78]. TRIM72 is a 53 kDa protein consisting of 477 amino acids. TRIM72 is composed of a RING domain, B-box domain, coiled helix domain, and PRY/SPRY domain [79]. TRIM72 is mainly expressed in the heart and skeletal muscles. TRIM72 is expressed in the kidneys, lungs, liver, and brain. TRIM72 has multiple functions, including a classic membrane repair function, anti-inflammatory ability, and E3 ligase activity [80]. TRIM72 controls the degradation of several substrate proteins, such as insulin receptor, IRS-1 [81], FAK [82], cyclin D1 [83], and Ras-related C3 botulinum toxin substrate 1 (RAC1) [84]. TRIM72 reportedly has a protective effect on multiple organs, including cardiac tissues. Previous studies have demonstrated that TRIM 72 alleviates ischemia/reperfusion injury, cardiac arrhythmia, heart failure, and cardiomyopathy [80]. Notably, treatment with human recombinant TRIM72 reportedly improves cardiac function in elderly mice by reducing oxidative stress and cardiomyocyte apoptosis [85].

A recent report has demonstrated the role of TRIM72 in DIC. The myocardial protein levels of TRIM72 were downregulated in mice treated with Dox. Myocardial-specific TRIM72 interacts with p53 and promotes the K48-linked poly-ubiquitylation and degradation of p53 in DIC, independent of MDM2. TRIM72-overexpressing mice showed preserved cardiac function and effectively reduced myocardial ferroptosis by increasing SLC7A11 levels in DIC. Thus, TRIM72 can protect against DIC by increasing p53 ubiquitylation, indicating that TRIM72 could be a new therapeutic target for DIC [86].

### 3.5. E6AP

E6-associated protein (E6AP) was first identified as a cellular protein that binds to the E6 oncoprotein from high-risk human papillomaviruses (HPV-16 and HPV-18), forming a complex that promotes p53 ubiquitylation and proteasomal degradation [87,88,89,90]. p53 degradation enhanced by this complex has been implicated in cervical carcinogenesis. In the presence of E6, E6AP undergoes self-ubiquitylation and degradation [91], which paradoxically increases p53 levels and activates the p53/MDM2 pathway [92]. The ligase activity of E6AP is generally modulated by E6; however, it is also regulated by several factors, such as the HIV-1 Nef protein in HIV [93] and HERC2 in Angelman syndrome [94,95,96]. We reported that diacylglycerol kinase (DGK) ζ directly binds to E6AP through ankyrin-like repeats and regulates E6AP activity in the heart [19]. The DGKζ enzyme catalyzes the phosphorylation of diacylglycerol to phosphatidic acid predominantly expressed in the heart [97]. It reportedly interacts with MDM2 and regulates the p53 protein expression level by inducing its ubiquitylation in HeLa and neurons [98,99]. DGKζ transgenic mice preserve cardiac function and improve the survival rate in DIC via the inhibition of p53 expression [19]. Therefore, E6AP may participate in p53 degradation during DIC.

### 3.6. ITCH

The ubiquitin E3 ligase ITCH was originally identified after a genetic analysis of a mutant mouse with aberrant immunological phenotypes and constant skin scratching [100]. ITCH belongs to the NEDD4 family of HECT-type E3 ligases. The WW domain recognizes the Pro-rich PPXY consensus sequence in substrate proteins, and the HECT domain attaches ubiquitin molecules to substrates [101]. Poly-ubiquitylated substrate proteins are degraded by the ubiquitin–proteasome system [102]. ITCH interacts with DVL family proteins and inhibits cardiac hypertrophy after pressure overload [103]. ITCH also interacts with TRAF6 and TAK1 in cardiomyocytes and improves cardiac function and survival rates in septic cardiomyopathy by suppressing the nuclear factor-kappa B pathway [104]. ITCH reportedly interacts with TXNIP and induces its proteasomal degradation, leading to ROS inhibition in DIC. Although the interaction between ITCH and p53 has never been described, ITCH suppressed the protein levels of p53 and cardiomyocyte apoptosis in DIC [105]. miR-34b/c decreases HL-1 cell viability and promotes the secretion of proinflammatory cytokines in Dox-induced myocardial cells through the ITCH/nuclear factor kappa B pathway [106]. CircITCH reportedly acts as a natural sponge for miR-330-5p, thereby upregulating Sirtuin 6 (SIRT6), Survivin, and Sarco/endoplasmic reticulum calcium ATPase 2a (SERCA2a) expression to alleviate DIC [107]. These reports support the finding that ITCH inhibits DIC through p53 inhibition.

### 3.7. CHIP

CHIP was first identified as a chaperone regulator that interacts with and modulates the stability of Hsp70 and Hsc70 [108]. It was later found to possess E3 ubiquitin ligase activity [109]. CHIP consists of a tetratricopeptide repeat (TPR) domain, a coiled-coil region, and a U-box domain. At the N-terminus, CHIP specifically binds to the C-terminal peptide of Hsp70/Hsp90 to regulate proteostasis [110,111]. A coiled-coil region with a helix–turn–helix-like structure is important for dimerization [112]. At the C-terminus, the U-box domain interacts with E2 ubiquitin-conjugating enzymes to mediate substrate ubiquitylation [113]. CHIP functions as a homodimer with an asymmetric structure. Crystallographic analysis revealed that in one protomer, the E2-binding site of the U-box was occluded because of contact with the TPR domain, rendering it inactive, whereas in the other protomer, the site was exposed, representing the active form [114]. This asymmetry is essential for the regulation of ubiquitin ligase activity.

CHIP ubiquitinates chaperone-bound proteins, including cystic fibrosis transmembrane-conductance regulator [115] and tau [116]. CHIP is also known to regulate many oncogenic proteins, including ErbB2 [117] and HIF1-α [118]. Furthermore, CHIP is involved in the regulation of tumor suppressor proteins, such as p53 [119], apoptosis-inducing factor [120], and interferon regulatory factor 1 [121], which play well-known roles in the regulation of tumor suppressor proteins.

CHIP is essential for the clearance of misfolded or unfolded proteins, maintenance of mitochondrial function, and regulation of stress-induced apoptosis [122]. It is highly expressed in cardiac tissues and exerts cardioprotective effects [123]. In murine myocardial infarction models, CHIP regulates p53 protein levels, reduces cardiomyocyte apoptosis [124], promotes angiogenesis through HIF1α/VEGF signaling, and suppresses both apoptosis and inflammation [125]. CHIP also forms a complex with Extracellular signal-regulated kinase 5 (ERK5), modulates inducible cAMP early repressor (ICER) expression, and reduces myocardial apoptosis [126]. A previous study showed that CHIP transgenic mice exhibited attenuated cardiac atrophy, dysfunction, inflammation, and oxidative stress after Dox injection. Mechanistically, CHIP directly promotes p53 degradation and SHP-1, leading to the activation of the ERK1/2 and STAT3 signaling pathways. Therefore, CHIP overexpression ameliorates DIC [127].

## 4. The Action of Cardioprotective Drugs via Ubiquitylation

Since the first report of severe DIC, numerous efforts have been devoted to exploring cardioprotective agents to ameliorate it. However, only one compound, dexrazoxane, has been used to prevent DIC [128]. Unfortunately, several reports have suggested that dexrazoxane interferes with the anti-cancer activity of Dox by inhibiting topoisomerase II [129]. Thus, the discovery of novel cardioprotective agents is desirable, which promote a selective reduction in DIC without decreasing the anti-cancer effect. Several other cardioprotective agents were also examined (Table 2). Interestingly, most of these depend on the interaction between p53 and MDM2 in DIC.

### 4.1. Dihydromyricetin

Flavonoids are considered attractive compounds due to their iron-chelating, antioxidant, and carbonyl reductase-inhibitory characteristics for mitigating DIC. Dihydromyricetin (DMY), also known as ampelopsin, is a flavonoid extracted from *Ampelopsis grossedentata*. The cardioprotective effects of DMY in ischemia–reperfusion injury, diabetic cardiomyopathy, and cardiac fibrosis caused by angiotensin II stimulation models have been reported [130,131,132]. Previous reports have shown that DMY ameliorates DIC through several mechanisms, such as the inhibition of the nucleotide-binding oligomerization domain-, leucine-rich repeat-, and pyrin domain-containing receptor 3 (NLRP3) [133] and the activation of the AMPK/mTOR pathway [134].

A previous report indicated that the cardioprotective and anti-cancer activities of DMY against Dox depend on the interaction between MDM2 and its substrate proteins. The apoptosis repressor with a caspase recruitment domain is an important anti-apoptotic factor possessing antagonistic properties of both intrinsic and extrinsic cell death pathways [135]. DMY reduces the protein expression of MDM2 in cardiomyocytes and U937 cells expressing p53. Interestingly, DMY targets the apoptosis repressor with a caspase recruitment domain in cardiomyocytes and inhibits DIC via the degradation of the MDM2-dependent apoptosis repressor with a caspase recruitment domain. Intriguingly, DMY exhibits a synergistic effect with Dox in its anti-cancer activity in a p53-dependent manner. DMY could be a potential cardioprotective agent for the clinical treatment of DIC owing to the dual action of DMY on DIC and anti-cancer capacity [136].

### 4.2. Licochalcone A

Licorice is one of the most famous traditional Chinese herbs. Licorice contains five cardioprotective materials, liquiritin, isoliquiritin, liquiritigenin, isoliquiritigenin, and licochalcone A, which can alleviate DIC by suppressing oxidative stress and mitochondrial damage [137]. Licochalcone A (Lico A), a flavonoid found in licorice, has multiple pharmacological activities that modulate oxidative stress, glycemia, inflammation, and lipid metabolism [138]. A bioinformatic analysis of licorice showed that Lico A is a regulator of p53 and the PI3K/AKT signaling pathway in DIC. Mice treated with Lico A showed a significant amelioration of DIC-related histopathological and electrocardiographic abnormalities in the heart. At the protein level, Lico A increased the phosphorylation of PI3K/AKT/MDM2, resulting in reduced p53 accumulation. Additionally, Lico A upregulated SLC7A11 and GPX4 expression. Lico A attenuates DIC by suppressing p53-mediated ferroptosis via the activation of PI3K/AKT/MDM2 signaling [139].

### 4.3. Resveratrol

Resveratrol, a natural antioxidant commonly found in grapes, red wine, and berries, is a small molecule that activates the longevity-related gene Sirtuin 1 (SIRT1) [140]. Mounting evidence has demonstrated the cardioprotective effect of resveratrol [141,142]. Ubiquitin-specific protease 7 (USP7) is a p53-de-ubiquitinating enzyme that stabilizes it [143].

Sin et al. reported that Dox reduces SIRT1 deacetylase activity and elevates USP7 expression, leading to increased p53 expression. Resveratrol ameliorated DIC; however, this effect was antagonized by sirtinol and EX527, which are SIRT1 inhibitors. Thus, resveratrol could be a potential agent for DIC prevention by inhibiting USP7-dependent p53 de-ubiquitylation [144].

### 4.4. Quercetin

Quercetin is an important dietary flavonoid present in several fruits and vegetables and possesses antioxidant, anti-inflammatory, and anti-cancer properties [145]. Quercetin scavenges ROS and inhibits cardiomyocyte apoptosis in ROS-induced cardiomyopathy [146] and is more effective than other flavonoids such as naringenin, pycnogenol, and trolox in protecting against daunorubicin-induced cardiotoxicity in H9c2 cells [147].

Bmi-1 is an early DNA damage response protein that accumulates at the DNA double-strand brake foci and promotes double-strand brake repair [148]. The polycomb group proteins BMI1 and RING1B/RNF2 form an active heterodimer E3 ligase that catalyzes the mono-ubiquitylation of histone H2A at Lysine 119. Bmi-1 is required for the DNA damage-induced ubiquitylation of histone H2A at Lysine 119, and the loss of Bmi-1 impairs the repair of DNA DSBs through homologous recombination [149]. Quercetin-suppressed Dox induces DNA double-strand breaks and maintains the DNA repair capacity of cardiomyocytes by upregulated Bmi-1 expression, accompanied by p53 suppression in DIC [150]. Several reports showed that quercetin reduced Dox-induced toxic side effects and protected cardiomyocytes from toxicity [151,152]. Therefore, quercetin is a potential candidate for the prevention of DIC.

### 4.5. Ganoderma Lucidum Polysaccharides

Ganoderma lucidum is a basidiomycete white-rot fungus that has been widely used traditionally in the treatment of a variety of human diseases in China for many decades. A variety of bioactive substances have been extracted from *Ganoderma lucidum*, and *Ganoderma lucidum* polysaccharides (GLPSs) have been shown to be the most important materials responsible for its bioactivity.

The transcription factor NF-E2-related factor 2 (Nrf2), which regulates the expression of antioxidant and detoxification genes, has cardioprotective properties [153]. MDM2 is an Nrf2 target gene. Previously, the elevated MDM2 expression induced by Nrf2 was reported to be related to the downregulation of p53, resulting in the inhibition of mitochondrial apoptosis [154].

GLPS treatment significantly attenuated Dox-induced histological changes in the heart tissue of rats treated with Dox. GLPS pretreatment markedly attenuated myocardial apoptosis, potentiated oxidative stress, and decreased the activity of antioxidant enzymes in Dox-treated H2C9 cells. Mechanistically, GLPS pretreatment stabilized Nrf2 expression by inhibiting the Cul3-mediated K48-linked poly-ubiquitylation of Nrf2 in Dox-treated H9c2 cells. Thus, GLPS attenuated DIC by decreasing p53 expression via MDM2 elevation [155].

### 4.6. FGF1 Variant

Fibroblast growth factor 1 (FGF1) is a member of the FGF family. Wild-type FGF1 (FGF1^WT^) reportedly possesses antioxidative and anti-apoptotic activities in various diseases [156]. Mounting evidence has demonstrated the cardioprotective effects of FGF1^WT^. However, the clinical application of FGF1^WT^ is limited due to the risk of tumorigenesis [157]. Thus, an FGF1 variant (FGF1^ΔHBS^) was engineered.

FGF1 is reportedly downregulated in DIC, as seen in the hearts of Dox-treated mice, primary cardiomyocytes, and H9c2 cells. Treatment with an FGF1 variant (FGF1^ΔHBS^) prevented cardiac dysfunction, inflammation, fibrosis, and hypertrophy. FGF1^ΔHBS^ treatment attenuated cardiomyocyte apoptosis and oxidative stress in DIC. Mechanistically, the cardioprotective effect of FGF1^ΔHBS^ was mediated by decreasing p53 activity through the upregulation of SIRT1-mediated p53 deacetylation and an enhancement in MDM2-mediated p53 ubiquitylation. Of note, the upregulation of p53 expression or cardiac-specific SIRT1 knockout abolished FGF1^ΔHBS^-related cardioprotective effects in DIC, suggesting that FGF1^ΔHBS^ depends on the SIRT1 and p53 axis. FGF1^ΔHBS^ could be a potential therapeutic agent against DIC [158].

### 4.7. Saussurea involucrata

*Saussurea involucrata*, a perennial herb of the family Compositae, is a popular medicinal plant in Xinjiang [159]. *Saussurea involucrata* injection is a sterile aqueous solution derived from dead aerial parts of *Saussurea involucrata* that has anti-inflammatory and antioxidative effects. *Saussurea involucrata* injection reportedly inhibits inflammation via the MAPK and NFκB pathways in rheumatoid arthritis [160].

A previous study examined the effect of *Saussurea involucrata* injections on DIC. *Saussurea involucrata* injection significantly improved Dox-induced cardiac dysfunction and reduced pathological alterations and fibrosis in cardiomyocytes. *Saussurea involucrata* injection exerts cardioprotective effects by diminishing inflammation, oxidative stress, and apoptosis triggered by Dox. Network pharmacological analysis showed that *Saussurea involucrata* injection downregulated p53 protein expression by activating the AKT/MDM2 signaling pathway. Therefore, *Saussurea involucrata* injection may be a potential therapy for preventing DIC [161].

### 4.8. Qishen Granule

Qishen granule is a traditional Chinese medicine formula. It consists of six herbs: Radix Astragali mongolici, Radix Salvia miltiorrhizabunge, Flos Lonicerae, Radix Scrophulariae, Radix Aconiti Lateralis Preparata, and Radix Glycyrrhizae. It was developed from the traditional formula “Zhen-Wu-Tang.” Qishen granules protect against ER stress-induced myocardial apoptosis via the inositol-requiring enzyme 1 (IRE1)–alpha B-Crystallin (CRYAB) pathway, which is a promising therapeutic target for myocardial ischemia [162].

Previous reports have shown that activated cytosolic p53 binds to Parkin and disrupts its translocation to damaged mitochondria and subsequent clearance by mitophagy in DIC [163].

Qishen granules protected against Dox-induced myocardial structural and functional damage, mitochondrial oxidative damage, and apoptosis. Dox inhibits mitochondrial biogenesis and blocks mitophagy in mouse myocardium, whereas Qishen granules reverse these effects. Qishen granules can promote the degradation of p53 by enhancing the binding of MDM2 to the p53 protein, resulting in the reduced binding of p53 to the Parkin protein, thus improving Parkin-mediated mitophagy. Qishen granules relieve Dox-induced mitochondrial oxidative damage and apoptosis by coordinating mitophagy and mitochondrial biogenesis [164].

## 5. Discussion

**Figure 2:**
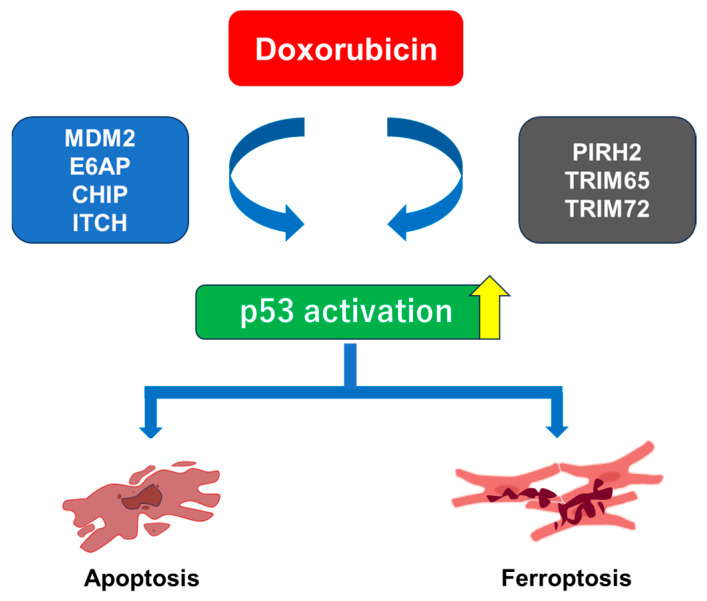
An integrative model illustrating the role of E3 ligases in Dox-induced cardiotoxicity.

As shown in Table 1 and Figure 2, recent reports demonstrated that several kinds of E3 ligases are involved in p53 degradation, and p53 suppression is of critical importance to mitigate DIC via apoptosis and ferroptosis. On the other hand, these reports did not shed light on the anti-cancer effects of E3 ligases simultaneously. Therefore, it is still unclear whether E3 ligase-mediated p53 suppression could be a therapeutic target for DIC without inhibiting the anti-cancer effects of E3 ligases. Thus, there is still room for discussion as to whether the mechanism described in this review could be applicable for the treatment of DIC.

As shown in Table 2, cardioprotective agents were examined in light of MDM2-dependent p53 suppression despite several E3 ligases degrading p53 in cardiomyocytes. Therefore, data are lacking about other E3 ligase-based cardioprotective agents. Since several E3 ligases are considered druggable targets [165,166], it is plausible that other E3 ligase activators or inhibitors could be anti-cancer and/or cardioprotective agents for DIC.

Similarly to research on the mechanisms of DIC, studies regarding cardioprotective agents for DIC should also be discussed in light of the dual effects of anti-cancer and cardioprotective properties. Zhu H et al. reported that DMY targets different substrates between cardiomyocytes and cancer cells. Interestingly, in cancer cells, the expression levels of p53 affect its efficacy [136]. This report suggested that the cardioprotective agent does not always affect cardiomyocytes and cancer cells via the same mechanism. Also, cardioprotective agents for the anti-cancer effect may differ by cancer type. In the future, when examining cardioprotective agents for DIC, an approach specifically focusing on cancer type may be warranted.

## 6. Conclusions

p53 has been proposed as an important therapeutic target to prevent DIC. The identification of cardioprotective agents that regulate p53 expression may provide a new avenue for preventing DIC. However, p53 activation is also essential for the anti-cancer efficacy of doxorubicin. Therefore, it is critical to develop strategies that protect the heart without compromising tumor suppression. Ubiquitin E3 ligases are often focal points of cellular regulation, making them attractive therapeutic targets. Recent experimental studies have emphasized the role of ubiquitin E3 ligases in DIC development through the regulation of p53 expression. Natural compounds that modulate E3 ligase-mediated p53 expression are attracting increasing attention. Future investigations should focus on the selective regulation of p53 signaling, for instance, by employing cardiac-specific delivery systems or by targeting post-translational modifications, as such approaches may enable cardioprotection while maintaining the anti-cancer efficacy of doxorubicin. Continued research examining the interactions between p53 and E3 ligases is critical to increase our knowledge and discover new therapeutic targets for the prevention of DIC.

## Figures and Tables

**Table 1:** E3 ligases reported to regulate p53 in doxorubicin cardiotoxicity at the cardiac/cardiomyocyte level.

E3 Ligase	Type	Experimental Model (In Vivo/In Vitro)	Downstream Effects
MDM2	RING	Mouse myocardium /H9c2 cells	Modulation of apoptosis and autophagy/mitophagy; cardioprotection under Dox stress
PIRH2	RING	Mouse myocardium /HL-1 cells	Inhibition of Dox-induced ferroptosis; improvement in cardiac function
TRIM65	RING	Mouse myocardium /H9c2 cells	Suppression of Dox-induced ferroptosis; myocardial protection
TRIM72	RING	Mouse myocardium /H9c2 cells, NRCM	Suppression of ferroptosis via p53/SLC7A11/GPX4 axis; cardioprotection
E6AP	HECT	Mouse myocardium /H9c2 cells, NRCM	Dgkζ–Hsp70 complex-mediated regulation; reduced apoptosis
ITCH	HECT	Cardiac-specific ITCH transgenic mice /NRCM	Degradation of TXNIP; reduced ROS production; suppression of apoptosis; improved cardiac function after Dox exposure or MI
CHIP	U-box	CHIP transgenic mouse myocardium /H9c2 cells, NRCM	Attenuation of Dox-induced cardiotoxicity; inhibition of apoptosis

MI, myocardial infarction; TXNIP, thioredoxin-interacting protein.

**Table 2:** Cardioprotective agents against Dox-induced cardiotoxicity.

Agents	Substrate	E3 Ligases/ De-Ubiquitinating Enzyme
Dihydromyricetin	ARC ↑	MDM2 ↓
Licochalcone A	p53 ↓	MDM2 ↑
Resveratrol	p53 ↓	USP7 ↓
Quercetin	p53 ↓ (indirect)	Bmi-1 ↑
Ganoderma lucidum polysaccharides	p53 ↓	MDM2
FGF1 variant	p53 ↓	MDM2
Saussurea involucrata	p53 ↓	MDM2
Qishen granule	p53 ↓	MDM2

ARC, apoptosis repressor with caspase recruitment domain. ↑ means upregulation. ↓ means downregulation.

## Data Availability

The data that support the fundings of this study are available from the corresponding author upon reasonable request.

## Abbreviations

The following abbreviations are used in this manuscript:

CHIP	Carboxyl terminus of Hsp70-interacting protein
DIC	Doxorubicin-induced cardiotoxicity
E6AP	E6-associated protein
FGF1	Fibroblast growth factor 1
GPX4	Glutathione peroxidase 4
HECT	Homologous to E6AP C-terminus
MDM2	Mouse double mutant 2 homolog
Nrf2	Transcription factor NF-E2-related factor 2
RBRs	RING-between-RINGs
RING	Really interesting new gene
TRIM	Tripartite motif family proteins
SIRT1	Sirtuin 1
SLC7A11	Solute carrier family 7 member 11
